# Genome and GWAS analysis identified genes significantly related to phenotypic state of *Rhododendron* bark

**DOI:** 10.1093/hr/uhae008

**Published:** 2024-01-10

**Authors:** Qiannan Ye, Lu Zhang, Qing Li, Yaliang Ji, Yanli Zhou, Zhenzhen Wu, Yanting Hu, Yongpeng Ma, Jihua Wang, Chengjun Zhang

**Affiliations:** Germplasm Bank of Wild Species, Yunnan Key Laboratory for Crop Wild Relatives Omics, Kunming Institute of Botany, Chinese Academy of Science, Kunming, Yunnan 650201, China; University of Chinese Academy of Sciences, Beijing 100049, China; Flower Research Institute of Yunnan Academy of Agricultural Sciences, National Engineering Research Center for Ornamental Horticulture, Yunnan Academy of Agricultural Sciences Kunming 650000, China; Germplasm Bank of Wild Species, Yunnan Key Laboratory for Crop Wild Relatives Omics, Kunming Institute of Botany, Chinese Academy of Science, Kunming, Yunnan 650201, China; University of Chinese Academy of Sciences, Beijing 100049, China; State Key Laboratory of Materials-Oriented Chemical Engineering, College of Biotechnology and Pharmaceutical Engineering, Nanjing Tech University, Nanjing 211800, China; Germplasm Bank of Wild Species, Yunnan Key Laboratory for Crop Wild Relatives Omics, Kunming Institute of Botany, Chinese Academy of Science, Kunming, Yunnan 650201, China; Germplasm Bank of Wild Species, Yunnan Key Laboratory for Crop Wild Relatives Omics, Kunming Institute of Botany, Chinese Academy of Science, Kunming, Yunnan 650201, China; University of Chinese Academy of Sciences, Beijing 100049, China; Germplasm Bank of Wild Species, Yunnan Key Laboratory for Crop Wild Relatives Omics, Kunming Institute of Botany, Chinese Academy of Science, Kunming, Yunnan 650201, China; Yunnan Key Laboratory for Integrative Conservation of Plant Species with Extremely Small Populations, Kunming Institute of Botany, Chinese Academy of Sciences, Kunming 650201, China; Flower Research Institute of Yunnan Academy of Agricultural Sciences, National Engineering Research Center for Ornamental Horticulture, Yunnan Academy of Agricultural Sciences Kunming 650000, China; Germplasm Bank of Wild Species, Yunnan Key Laboratory for Crop Wild Relatives Omics, Kunming Institute of Botany, Chinese Academy of Science, Kunming, Yunnan 650201, China; Haiyan Engineering & Technology Center, Zhejiang Institute of Advanced Technology, Jiaxing 314022, China; State Key Laboratory of Subtropical Silviculture, Zhejiang A&F University, Hangzhou 311300, China

## Abstract

As an important horticultural plant, *Rhododendron* is often used in urban greening and landscape design. However, factors such as the high rate of genetic recombination, frequent outcrossing in the wild, weak linkage disequilibrium, and the susceptibility of gene expression to environmental factors limit further exploration of functional genes related to important horticultural traits, and make the breeding of new varieties require a longer time. Therefore, we choose bark as the target trait which is not easily affected by environmental factors, but also has ornamental properties. Genome-wide association study (GWAS) of *Rhododendron delavayi* (30 samples), *R. irroratum* (30 samples) and their *F*_1_ generation *R. agastum* (200 samples) was conducted on the roughness of bark phenotypes. Finally, we obtained 2416.31 Gbp of clean data and identified 5 328 800 high-quality SNPs. According to the *P*-value and the degree of linkage disequilibrium of SNPs, we further identified 4 out of 11 candidate genes that affect bark roughness. The results of gene differential expression analysis further indicated that the expression levels of *Rhdel02G0243600* and *Rhdel08G0220700* in different bark phenotypes were significantly different. Our study identified functional genes that influence important horticultural traits of *Rhododendron*, and illustrated the powerful utility and great potential of GWAS in understanding and exploiting wild germplasm genetic resources of *Rhododendron*.

## Introduction

*Rhododendron*, containing over 1000 species, is one of the largest genera in the family Ericaceae [1]. As the center of *Rhododendron* diversity, distribution and differentiation [2], China has 720 species of wild *Rhododendron* in seven subgenera, of which 450 species are endemic. Known as the ‘king of woody flowers’, rhododendrons are often used in urban greening and garden landscape design because of their beautiful shape and gorgeous flower colors [3].

*Rhododendron delavayi*, *R. irroratum* and *R. agastum* all belong to subgenus *Hymenanthes* (H. Sleumer and A. Gray). Among them, *R. delavayi* is a vigorous and graceful tree with large, bright red, long-flowering flowers, and is strongly adaptable to harsh environments, e.g. it is resistant to cold and drought. Practically, *R. delavayi* is widely used in landscaping as a high-grade potted flower as well as an important parent for the breeding of new *Rhododendron* cultivars [4]. In contrast, *R. irroratum* has smaller, creamy white or yellow flowers with attractive markings in the corolla, though it also is strongly resistant to cold conditions [5] and used as a parental plant for the breeding of new cultivars. *Rhododendron agastum* was originally described as a species but has since been demonstrated to be a hybrid of *R. delavayi* and *R. irroratum* [4]. These three *Rhododendron* taxa occupy different ecological niches along elevation gradients, forming a hybrid belt on the vertical gradient.

Genome-wide association studies (GWAS) are a useful method to identify genes related to important morphological traits in plants. Utilizing high-density single-nucleotide polymorphisms (SNPs), GWAS has greatly improved marker resolution and revolutionized the genetic dissection of complex trait architectures in many important crop species and horticultural plants [6, 7]. For example, several candidate loci related to important fruit quality traits, such as *ClTST2*, related to sugar accumulation and transport, were identified in watermelon from GWAS based on resequencing of germplasm resources [8]. Similarly, using combined GWAS and quantitative trait locus (QTL) mapping, the core markers of plum weeping traits were identified [9]. Sixteen million SNPs were called from resequencing of cotton cultivars and several cotton quality traits were identified from subsequent GWAS. For example, the roles of five candidate genes in four key traits in cotton—disease resistance, fiber length, fiber strength, and lint rate—were determined [10]. Recently, based on a high-quality peach genome assembly, GWAS was used to analyze convergent selection of sweetness and the differentiated selection of acidity between the East and the West during peach domestication. Specifically, two genes, *PpALMT1* and *PpERDL16*, involved in the accumulation of organic acids and soluble sugars in fruits have been elucidated [11]. Utilizing single-base resolution of whole-genome genetic variants in *Vitis*, selective sweeping was found to improve both the edibility and stress resistance of berries, and associations between candidate genes and important agronomic traits, such as berry shape and aromatic compounds, were found [12].

The increasing amount of available genomic data has laid a solid foundation for our work on GWAS in *Rhododendron*. Most of the species in subgenus *Hymenanthes* of *Rhododendron* are diploid (2*n* = 2*x* = 26). *Rhododendron delavayi* was the first *Rhododendron* genome sequenced, with a genome size of 695.09 Mb [13]. Since then, several *Rhododendron* genomes have been published [14–16].

In this study we conducted a GWAS study of three *Rhododendron* taxa as a case study to identify key functional genes that affect the formation of important phenotypes. We used high-throughput resequencing data from *R. delavayi*, *R. irroratum*, and *R. agastum* to align a higher-quality *R. delavayi* reference genome for variant calling [14]. We performed population genetics and GWAS analyses to dissect the allele frequency spectra of the three taxa and further to establish the association of genotypes and phenotypes. Finally, we identified four genes that were significantly related to bark roughness among three taxa.

## Results

### Genomic variation, linkage disequilibrium and collinearity analysis

**Figure 1:**
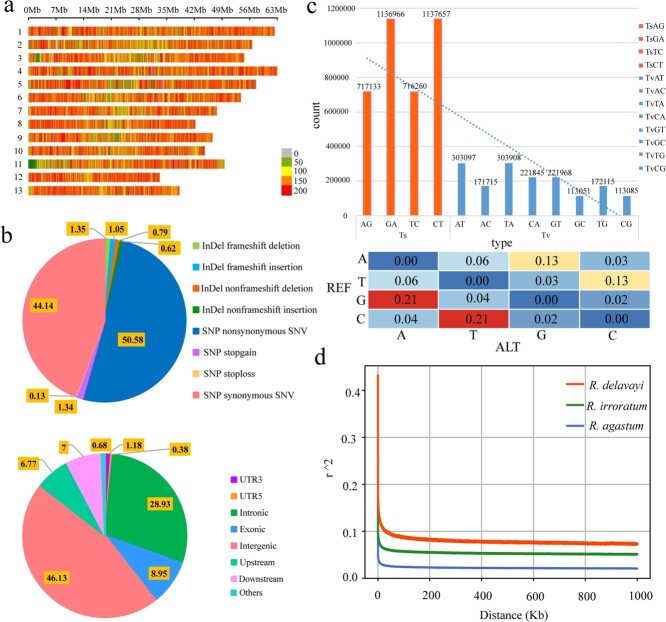
SNP density distribution on chromosomes, proportion of different variants and their location distribution in the genome, and general statistics pertaining to the number of different base substitutions, LD decay, and collinearity analysis between *R. delavayi* and *R. irroratum*. **a** Density distribution of 5 328 800 high-quality SNPs on 13 chromosomes in *Rhododendron* with MAF >0.05 and missing rate ≤10%. **b** Statistics of different variant sites and their locations on the genome. Using the *R. delavayi* genome as the reference genome, the variant sites were annotated and quantified using ANNOVAR. **c** Different types of base substitution at variant sites. **d** Genome-wide average LD decay in *R. delavayi*, *R. irroratum*, and *R. agastum*.

We generated high-quality resequencing data for 260 *Rhododendron* samples from Baili Rhododendron Nature Reserve. We obtained 8067.62 Mb clean reads and 2416.31 Gbp clean data, with an average Q30 of 92.83% (Supplementary Data Table S1). The *R. delavayi* genome (662.80 Mb) was used as the reference genome and the samples had an average sequencing depth of 7.7-fold (Supplementary Data Table S2). After multi-level filtering, 258 individuals were retained, and 5 328 800 high-quality SNPs were identified on 13 chromosomes (Fig. 1a). The obtained SNP data sets have been uploaded to the website RPGD and are available for download (http://bioinfor.kib.ac.cn/RPGD/). The number of SNPs per chromosome ranged from 339 011 on chromosome 12 to 495 188 on chromosome 4. SNPs averaged 124.0 bp per SNP and varied from 99.0 bp on chromosome 12 to 145.0 bp on chromosome 2 (Supplementary Data Table S3). This average density is quite large when compared with other domesticated species, such as rice, where the average density is ~2.62 kb per SNP [17]. The higher SNP density may be a result of the spatiotemporal evolution of the *Rhododendron* taxa studied. All variable sites were divided into eight categories (Fig. 1b), the largest proportion being non-synonymous single-nucleotide variants (50.58%), followed by synonymous single-nucleotide variants (44.14%), frameshift deletions (1.35%), stopgains (1.34%), frameshift insertions (1.05%), non-frameshift deletions (0.79%), non-frameshift insertions (0.62%), and stoplosses (0.13%). SNPs were mainly distributed in intergenic and intronic regions, accounting for 46.13 and 28.93% of all SNPs, respectively, and SNPs distributed in exon regions accounted for 8.95% of the total SNPs (Fig. 1b). The transition/transversion ratio (Ts/Tv) was 2.29, which was greater than the critical value of 2 (Fig. 1c). These results indicated that the nucleotide substitutions of the three *Rhododendron* taxa were mainly transitions and that the locus variation in the genome did not reach saturation state. Linkage disequilibrium (LD) analysis of genome-wide SNPs identified 875 983 LD blocks (Supplementary Data 1), of which 48.91% had only two SNPs (428 454 LD blocks). LD decay patterns of the three subgenomes were basically the same, and decreased sharply within the first 0–2 kb and dropped to the minimum mean_*r*^2^ before the distance had reached 2 kb (Fig. 1d). Of the three *Rhododendron* taxa studied, *R. agastum* had the fastest LD decay rate, and the physical distance on the chromosomes when the LD decay rate dropped to half its maximum value (mean_*r*^2^: 0.08) was only 64 bp. The chromosomal physical distances in *R. delavayi* and *R. irroratum* were 150 bp (mean_*r*^2^: 0.22) and 132 bp (mean_*r*^2^: 0.12), respectively when LD had dropped to half the maximum value. These results suggest that *R. agastum* has higher genetic diversity than either *R. delavayi* or *R. irroratum*.

### Analysis of *Rhododendron* population structure and kinship

**Figure 2:**
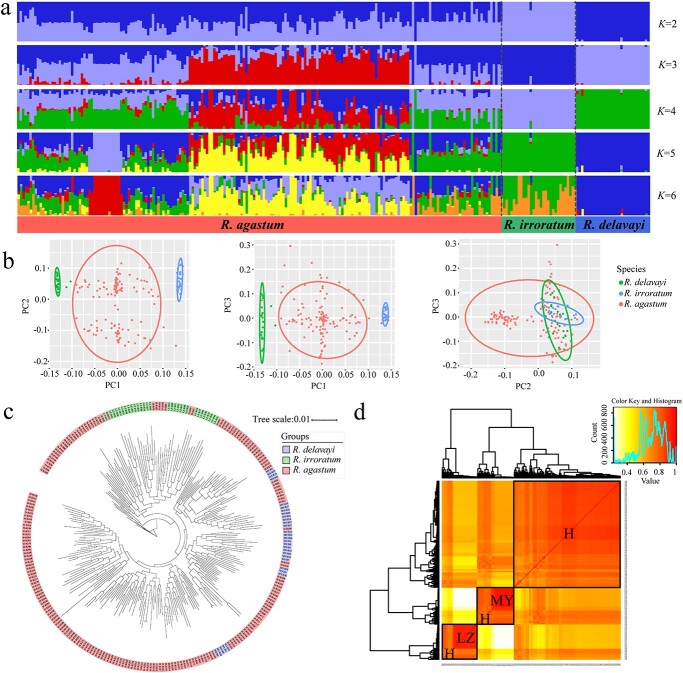
Population structure, PCA, phylogenetic neighbor-joining tree and kinship analyses. **a** Population structure analysis results for *K* = 2–6. The *y*-axis quantifies cluster membership, and the *x*-axis represents different accessions. **b** PCA plot of the first three components for three *Rhododendron* taxa. **c** Phylogenetic neighbor-joining tree based on 260 *Rhododendron* samples. **d** IBS analysis of the three studied *Rhododendron* taxa. The samples framed in black are those with high sequence similarity among all three *Rhododendron* species, and are more closely related than other samples.

Population structure was described by gene frequency and genotype frequency. We investigated cross-validation errors for *K* = 1–10, and found that the cross-validation error at *K* = 3 was minimal (Supplementary Data Fig. S1), indicating the presence of three major gene pools, and allowing the 260 individual *Rhododendron* genotypes to be divided into three groups (Fig. 2a). Each genotyped individual was assigned to a designated population using Q ≥ 0.70 as a criterion for division. A total of 135 individuals were assigned to three groups, of which 45 individuals were assigned to pop1, 42 individuals to pop2, and 48 individuals to pop3, and the remaining 123 individuals to mixed groups. The number of individuals belonging to pop1 accounted for 17.44% of the total number of statistical samples, and included 30 *R. delavayi* and 15 *R. agastum* individuals. The number of individuals belonging to pop2 accounted for 16.28% of the total number of statistical samples, and included 30 *R. irroratum* and 12 *R. agastum* individuals. The number of individuals belonging to pop3 and the mixed populations accounted for 18.60 and 47.67% of the total number of statistical samples, respectively, all of which were *R. agastum* individuals. These results were consistent with those of the principal component analysis (PCA) (Fig. 2b) and the phylogenetic tree (Fig. 2c). PLINK was used for PCA analysis and when —pca was set as 10, the cumulative contribution rate of the first two principal components was 64.08%, with PC1 and PC2 explaining 58.79 and 5.29% of the total variation, respectively. When the parameter was changed to 3, the cumulative contribution of the first two principal components was 91.33%, with PC1 and PC2 explaining 76.64 and 14.69% of the total variation, respectively (Supplementary Data Fig. S2).

Identity-by-state (IBS) analysis was used to analyze the same number of alleles in the same locus (loci) between different individuals, and the genetic distance matrix was constructed. The genetic distance between the three *Rhododendron* species was analyzed (Fig. 2d; Supplementary Data 2). These results indicated that there was obvious stratification of population structure among the three *Rhododendron* taxa. *Rhododendron delavayi* and *R. agastum* were more closely related, while *R. irroratum* and *R. agastum* had a more distant relationship.

### Asymmetric selection signatures among the three *Rhododendron* taxa

**Figure 3:**
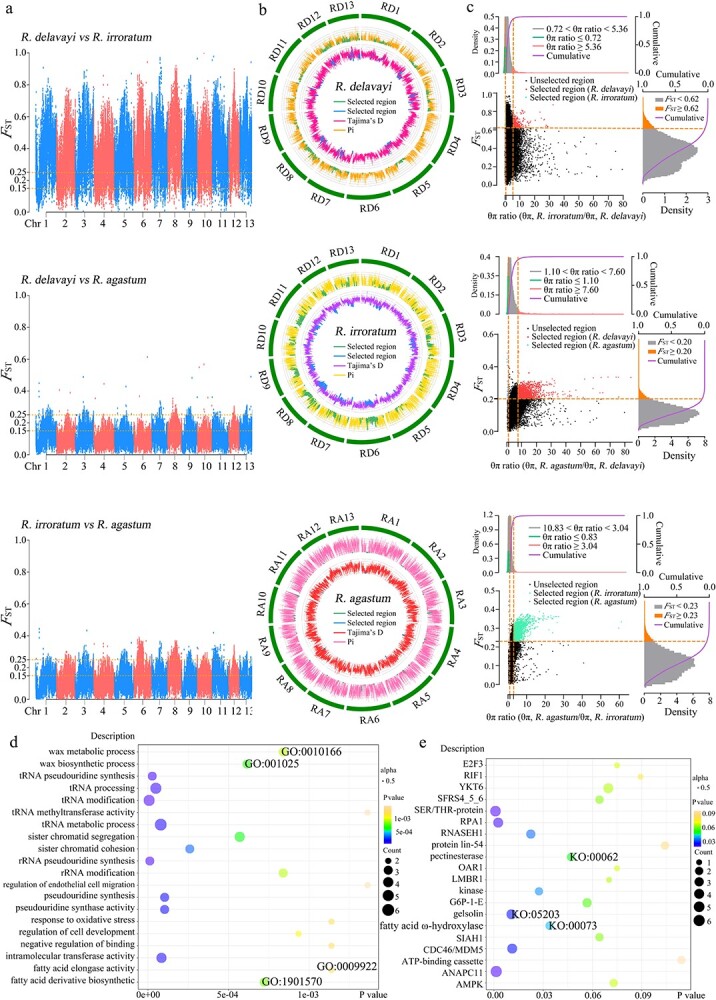
Detection of regions under natural selection in the genomes of the three studied *Rhododendron* taxa; selective clearance assays for the three *Rhododendron* taxa and GO and KEGG enrichment analyses of genes in regions under selection in the genomes of the three studied *Rhododendron* taxa. **a** Degree of population differentiation between *R. delavayi* and *R. irroratum*, *R. delavayi* and *R. agastum*, and *R. irroratum* and *R. agastum*. The black threshold line indicates an *F*_ST_ value of 0.15 and the red threshold line indicates an *F*_ST_ value of 0.25. **b***Rhododendron delavayi*, *R. irroratum*, and *R. agastum* selective sweep analysis results. Regions of loci under selection were identified using the first 5% *F*_ST_ and the genetic diversity (θπ) ratio. The red, green, and blue areas represent regions under selection in the genomes of *R. delavayi*, *R. irroratum*, and *R. agastum,* respectively. **c** Nucleotide diversity (Pi) and neutrality detection (Tajima’s *D*) of the three studied *Rhododendron* taxa. The sliding window is 50 000 bp. **d** GO enrichment analysis of *R. delavayi*, *R. irroratum*, and *R. agastum* genes in regions under selection. **e** KEGG enrichment analysis of *R. delavayi*, *R. irroratum*, and *R. agastum* genes in regions under selection.

Our analysis demonstrated that there was a high degree of genetic differentiation between *R. delavayi* and *R. irroratum*. The *F*_ST_ of *R. delavayi* and *R. irroratum* was statistically analyzed, and a total of 129 401 intervals were obtained, with the mean *F*_ST_ value of the interval being 0.36. This indicated that there was no comprehensive gene flow between *R. delavayi* and *R. irroratum*, and there was a large genetic differentiation between the two subpopulations. The *F*_ST_ values of *R. delavayi* and *R. agastum*, and of *R. irroratum* and *R. agastum* were lower than that of *R. delavayi* and *R. irroratum*. The *F*_ST_ value of *R. delavayi* and *R. agastum* ranged from 0 to 0.61, with a total of 129 650 intervals and with the mean *F*_ST_ value being 0.11. The mean *F*_ST_ value of *R. irroratum* and *R. agastum* was 0.12, with a total of 129 771 intervals obtained (Fig. 3a; Supplementary Data 3–5; Supplementary Data Table S4). Through the analysis and comparison of the *F*_ST_ values of the three *Rhododendron* taxa, we found obvious differentiation between *R. delavayi* and *R. irroratum*.

By comparing and analyzing genetic diversity (θπ) and *F*_ST_ values among different *Rhododendron* taxa, we identified 6624 regions under natural selection in the *Rhododendron* genome (Supplementary Data 6–8). There were 2514 selected regions in *R. delavayi*, the most highly selected region being region 423 on chromosome 8. *Rhododendron irroratum* had 3986 regions under selection, the most highly selected region being region 834 on chromosome 8. *Rhododendron agastum* had 124 regions under selection, the most highly selected region being region 17 on chromosome 1. Tajima’s *D* and nucleotide polymorphism suggested that the observed heterozygosity of *R. agastum* was higher than the expected heterozygosity. The mean value of Tajima’s *D* for *R. agastum* was 2.47, indicating that the *R. agastum* population contracted or was subject to equilibrium selection. The mean values of Tajima’s *D* for *R. delavayi* and *R. irroratum* were 0.28 and 0.78, respectively. Both of these values are close to 0, indicating that the observed heterozygosity of *R. delavayi* and *R. irroratum* was not very different from the expected heterozygosity, that the populations have evolved under mutation–drift equilibrium, and that pressure from natural selection was relatively low (Fig. 3b; Supplementary Data 9–11).

GO and KEGG enrichment analyses were performed on the genes in the top 5% of θπ*_R. irroratum_*/θπ*_R. delavayi_*, θπ*_R. agastum_*/θπ*_R. delavayi_*, θπ*_R. agastum_*/θπ*_R. irroratum_* and *F*_ST_ in the three studied *Rhododendron* taxa (Fig. 3c; Supplementary Data 12 and 13). GO and KEGG enrichment analyses of *R. delavayi*, *R. irroratum*, and *R. agastum* genes in the 1363 regions under selection found that genes with functions involved in ‘tRNA modification’, ‘rRNA pseudouridine synthesis’, ‘wax biosynthetic process’, and ‘fatty acid derivative biosynthetic process’ were significantly enriched. The KEGG analysis indicated that these genes were also involved in the synthesis of important functional substances in the processes of ‘cell mitosis’, ‘protein phosphatase dephosphorylation’, ‘DNA replication initiation and elongation’, ‘apoptosis’, and ‘lipid metabolism’ (Fig. 3d and e).

### Four candidate genes were identified by GWAS

**Figure 4:**
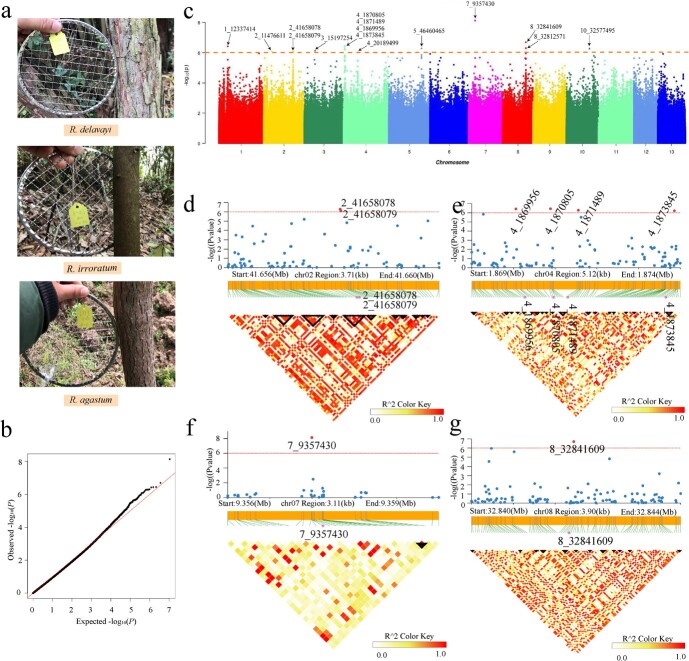
Bark phenotype, genome-wide association analysis of bark phenotypes and genotypes in three *Rhododendron* taxa, and degree of linkage of SNPs in genes significantly associated with bark phenotype. **a** Bark of *R. delavayi*, *R. irroratum*, and *R. agastum*. **b** QQ plot of association between bark phenotype and *Rhododendron* genotype. The selected correlation model was a mixed linear model, and the results from the analyses of population structure and kinship were used as the Q and K matrixes, respectively. **c** Manhattan plot of bark phenotype–genotype association results for the three studied *Rhododendron* taxa. The threshold was set to −log10(*P*) >6, and the significantly associated loci are marked with the location of the SNPs on the chromosome. **d**–**g** LD block of the four identified genes significantly associated with the bark phenotype. The degree of linkage between the SNPs was measured by the *r*^2^ value.

We sampled branches of different growth years and made observations on them. The bark of perennial branches of *R. delavayi* is rough, while the bark of the twigs of *R. delavayi*, the perennial branches and the twigs of *R. irroratum* is smooth.(Fig. 4a). The bark roughness of 260 samples was evaluated statistically and we found that the bark of *R. delavayi* was very rough, with crevices and many cracks, while the bark of *R. irroratum* was smooth and there were no obvious cracks. The bark of the wild hybrids *R. agastum* had characteristics of both *R. delavayi* and *R. irroratum* (Supplementary Data Table S5). We used a total of 5 328 800 high-quality SNPs for genome-wide association analysis of bark [missing rates ≤0.05, minor allele frequency (MAF) ≥ 0.05]. After threshold values were determined according to Bonferroni correction, 15 candidate sites were identified that were significantly associated with the target trait (Table 1). The frequencies of 15 SNPs identified by GWAS in different phenotypes were calculated. It was found that the frequencies of the 15 SNPs mentioned above were significantly different between *R. delavayi* and *R. irroratum* except for SNP 2_11476611 (Supplementary Data Table S6). Candidate sites were further reduced by comparing the *P*-value. Finally, the most significant association with target trait and multiple SNPs corresponding to the same gene were selected as the target SNPs. These SNPs correspond to four genes on chromosomes 2, 4, 7, and 8, respectively (Fig. 4b and c). BLASTX was used to compare the proteins encoded by the candidate regions with protein databases. The protein encoded by *Rhdel02G0243600* (region: Chr2:41655409–41665545 bp) was predicted to be peptidyl-prolyl *cis*-trans isomerase (FKBP17–1), that encoded by *Rhdel04G0017100* (region: Chr4:1869108–1881049 bp) was predicted to be transcription complex subunit 9-like (CCR4-NOT), and those encoded by *Rhdel07G0079700* (region: Chr7:9343765–9345531 bp) and *Rhdel08G0220700* (region: Chr8:32844048–32848852 bp) were predicted to be aspartic protease (AED3-like) and esterase/lipase protein (GDSL), respectively. The degree of SNP linkage on these four genes was analyzed. In *Rhdel02G0243600*, 35 LD blocks were detected, and the largest block covered 0.71 kb (Chr2:41659537–41660250) and contained 10 SNPs. The sites (2_41658078 and 2_41658079) with significant correlation with bark roughness were located in a block covering 0.39 kb (Chr2:41657790–41658181) and contained seven SNPs (Fig. 4d). *Rhdel04G0017100* contained 40 LD blocks, the largest of which covered 0.67 kb (Chr4:1870858–1871523) and contained six SNPs. With the exception of 4_1873845, the significantly associated loci were not in the LD block, and LD blocks 4_1869956, 4_1870805, and 4_1871489 covered 0.06, 0.17, and 0.67 kb, respectively (Fig. 4e). *Rhdel07G0079700* had the shortest gene sequence among the four candidate genes, and the number of LD blocks was also smallest of the four genes, with only nine LD blocks (Fig. 4f). The LD block with the largest coverage contained three SNPs and covered 0.26 kb (Chr7:9360606–9360861). SNP 7_9357430, which was significantly related to degree of bark roughness, was not located in the LD block (Fig. 4g). *Rhdel08G0220700* contained 36 LD blocks. The LD block with the largest coverage contained 13 SNPs and covered 0.76 kb (Chr8:32845484–32846239). SNP 8_32841609 was not in the LD block.

**Table 1:** Significant gene information related to bark phenotype.

	**SNP ID**	**Chromosome**	**Position**	***P* value**	**Gene ID**	**Coding protein name**
1	1_12337414	1	12337414	3.73E−07	*Rhdel01G0076300 Rhdel01G0076400*	YABBY5
2	2_11476611	2	11476611	8.35E−07	*Rhdel02G0081400*	UPF0481
3	2_41658078	2	41658078	8.17E−07	*Rhdel02G0243600*	FKBP17–1
4	2_41658079	2	41658079	8.17E−07		
5	3_15197254	3	15197254	7.37E−07	*Rhdel03G0099400 Rhdel03G0099500*	PPR
6	4_1870805	4	1870805	3.65E−07	*Rhdel04G0017100*	CCR4-NOT
7	4_1871489	4	1871489	5.34E−07		
8	4_1873845	4	1873845	6.23E−07		
9	4_1 869 956	4	1869956	3.84E−07		
10	4_20189499	4	20189499	9.34E−07	*Rhdel04G0139300 Rhdel04G0139400*	FAD2
11	5_46460465	5	46460465	5.23E−07	*Rhdel05G0258100*	UBP12
12	7_9357430	7	9357430	7.36E−09	*Rhdel07G0079700 Rhdel07G0079800*	AED3
13	8_32841609	8	32841609	2.02E−07	*Rhdel08G0220700*	GDSL
14	8_32812571	8	32812571	5.03E−07	*Rhdel08G0220100 Rhdel08G0220200*	DOF5.6
15	10_32577495	10	32577495	5.36E−07	*Rhdel10G0194200 Rhdel10G0194300*	LECRK3

When the threshold was set as 1.0 × 10^−5^, 109 loci significantly correlated with degree of bark roughness were identified. Four out of 109 loci were therefore located in genomic regions of the three *Rhododendron* taxa under selective pressure (6_15313273, 11_8986378, 11_8986396, and 12_17095967). The gene sequences of the significantly associated loci were extracted and compared with those in the NR library in NCBI. *Rhdel11G0024200* (11_8986378, 11_8986396) and *Rhdel12G0120600* (12_17095967) were predicted to encode polyadenylate binding protein (PABP) and agamous-like MADS-box protein, respectively. These two proteins have been reported to play important roles in the integrated stress response and in the regulation of floral organ and meristem development, respectively [18, 19].

### Candidate genes predicted to have functions related to bark development

**Figure 5:**
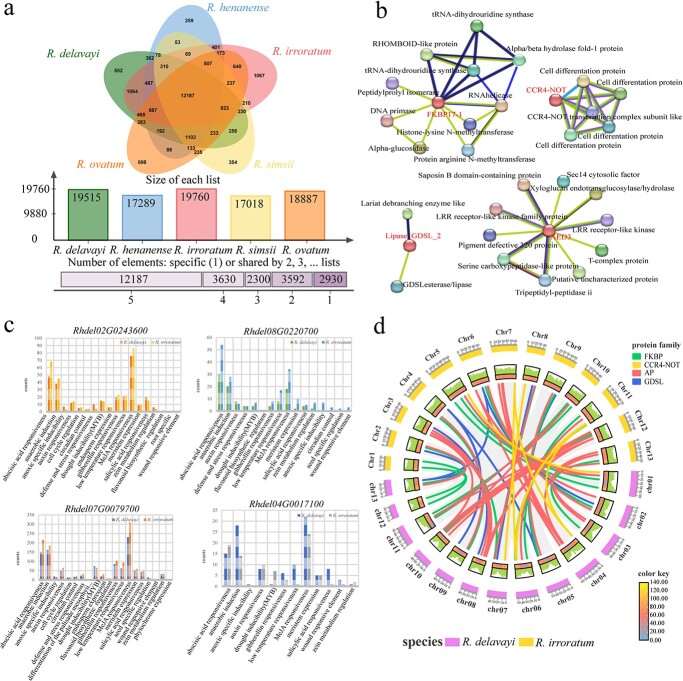
Analysis of gene family expansion and contraction in 10 species including *Rhododendron* and allied species, promoter type statistics, protein interaction network analysis of candidate genes, and genomic collinearity analysis of the candidate gene families in *R. delavayi* and *R. irroratum*. **a** Analysis of gene family contraction and expansion in 10 species, using *P. alba* and *V. vinifera* as outgroups. Venn diagram of homologous genes in five different *Rhododendron* species. **b** Proteins encoded by four candidate genes and the proteins that functionally interact with them. **c** Numbers of putative promoter elements in the four candidate genes. **d** Collinearity analysis of the candidate gene families in the genomes of *R. delavayi* and *R. irroratum*.

The results of our gene tree analysis showed that the gene families of the candidate genes were mainly formed by tandem duplication and segment duplication (Supplementary Data Figs S3–S6). To further explore the genetic changes behind the significant differences in bark phenotypes of our three studied *Rhododendron* taxa, and the evolutionary pressures that caused these changes, we used *Populus alba* and *Vitis vinifera* as outgroups, as well as five *Rhododendron* species (*R. delavayi*, *R. irroratum*, *R. simsii*, *R. ovatum*, and *R. henanense*) and the allied species *Camellia lanceoleosa*, *Vaccinium darrowii*, and *Actinidia chinensis* in an analysis of the expansion and contraction of gene families (Supplementary Data Fig. S7). The number of gene families showing significant expansions and contractions was 314 and 90, respectively, in *R. delavayi*, and 360 and 73, respectively, in *R. irroratum*. There were 12 187 gene families common to the five *Rhododendron* species (Fig. 5a; Supplementary Data Table S7).

The protein sequences encoded by candidate genes were uploaded to STRING for protein interaction network analysis, which suggested that the proteins interacting with the candidate proteins were mostly related to the maintenance of cell wall integrity and mRNA degradation (Fig. 5b). The proteins interacting with *Rhdel02G0243600* were found to be α/β hydrolase fold-1 protein, glucosidase, and RNA helicase. The proteins interacting with that encoded by *Rhdel04G0017100* were CCR4-NOT transcription complex subunit and cell differentiation protein. The proteins with high degrees of interaction with that encoded by *Rhdel07G0079700* were saposin B domain-containing protein and xyloglucan endotransglucosylase/hydrolase (XTH). The proteins interacting with that encoded by *Rhdel08G0220700* were lariat debranching enzyme and GDSL esterase/lipase. β-Glucosidase is believed to effectively eliminate the inhibitory effect of cellobiose produced during the process of enzymatic hydrolysis of cellulase and improve the efficiency of cellulose degradation [20]. XTHs are cell wall remodeling enzymes that can catalyze the breaking and reconnecting of xyloglucan molecules in the primary plant cell wall, and are responsible for the relaxing and strengthening of the cell wall, as well as participating in its degradation and synthesis [21].

From the promoter sequence results obtained by using PlantCARE, the functional types of promoters mainly focus on the regulation of the cell cycle, specific expression of proteins in various tissues, synthesis of substances such as flavonoids and zeatin, etc. The largest numbers of promoters in our four candidate genes were related to plant responses to methyl jasmonate or abscisic acid, and those under anaerobic induction (Fig. 5c). Recent studies have shown that methyl jasmonate and abscisic acid are involved in the regulation of plant secondary cell wall deposition and the synthesis of cellulose and lignin [22–24]. We obtained a total of 82 syntenic blocks through collinear analysis of the gene families of the four candidate genes. The largest numbers of syntenic blocks were found on chromosome 2 of *R. delavayi* and chromosome 6 of *R. irroratum,* with 15 syntenic blocks in both cases, and the coverage of the largest syntenic blocks was 9.4 kb (*Rhdel02G0186200*: 28219151–28229269, *Rhododendron_irroratum061080.1*: 28839793–28849239) (Fig. 5d).

### Transcriptome analysis and RT–qPCR in two *Rhododendron* species with different bark types

**Figure 6:**
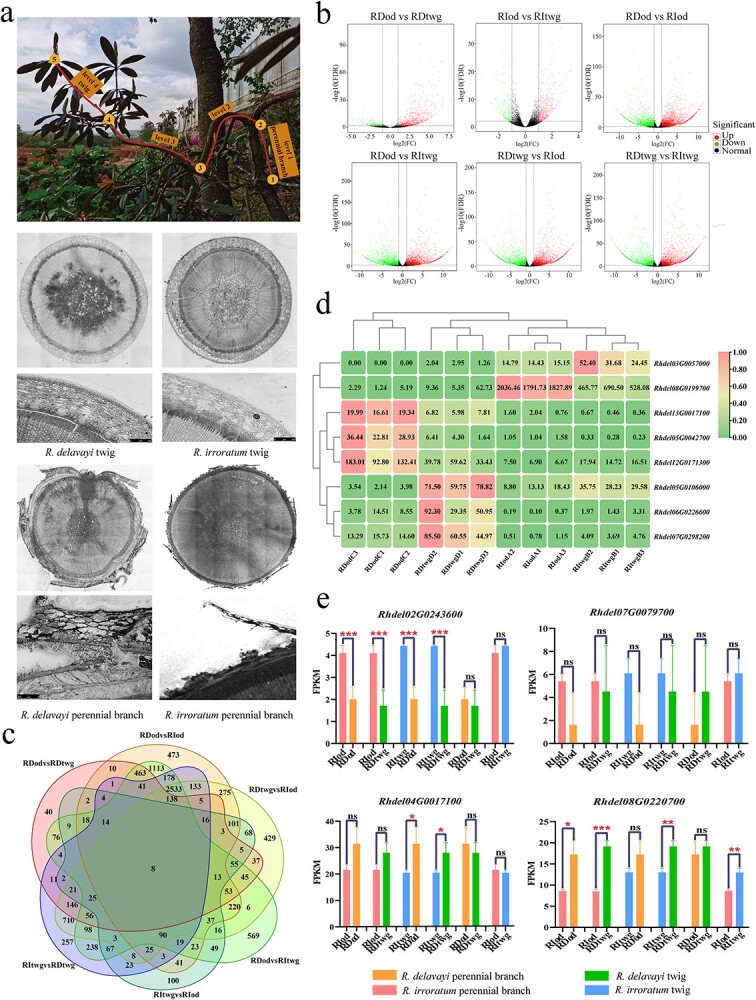
Sampling position diagram of *Rhododendron* and cross-section of *Rhododendron* branch anatomy, volcano plot of gene expression in different groups, Venn diagram of differential gene expression in different groups, expression heat map of significantly differentially expressed genes, and DEG analysis of candidate genes in different types of *Rhododendron* bark. **a** The upper part of the picture shows sampled plants. *Rhododendron* branches are divided into four levels according to how far away they are from the base of the plant. Nodes 1–2 are sampling perennial branches and nodes 4–5 are sampling twigs. The lower part of the image shows cross-sections of branch anatomy in *R. delavayi* and *R. irroratum*. **b** Differentially expressed genes between pairs of *R. delavayi* twigs, *R. delavayi* perennial branches, *R. irroratum* twigs, and *R. irroratum* perennial branches. **c** Venn diagram of gene expression levels in different groups. RDtwg, *R. delavayi* twigs; RDod, *R. delavayi* perennial branches; RItwg, *R. irroratum* twigs; RIod, *R. irroratum* perennial branches. **d** Expression heat map of eight DEGs among *R. delavayi* twigs, *R. delavayi* perennial branches, *R. irroratum* twigs, and *R. irroratum* perennial branches. **e** Differential gene expression analysis of four candidate genes in different types of *Rhododendron* bark.

Perennial branches and twigs of *R. delavayi* and *R. irroratum* were dissected. A cross-sectional view of each of the branches from *R. delavayi* and *R. irroratum* was obtained (Fig. 6a; Supplementary Data Fig. S8), as well as 61.79 Gb of clean sequencing data. The samples were divided into six groups according to the age of the branches (in growth years) and the differences in gene expression levels between different groups were compared. Fewest (986) differentially expressed genes (DEGs) were found between the group containing *R. irroratum* perennial branches and that containing *R. irroratum* twigs, and the largest number (8547) of DEGs was found between the groups containing *R. delavayi* perennial branches and *R. irroratum* twigs (Supplementary Data Table S8). The largest difference between the up- and downregulated genes was between the group of *R. delavayi* perennial branches and that of *R. delavayi* twigs, with 966 upregulated genes and only 160 downregulated genes (Fig. 6b). KEGG analysis of DEGs revealed that the most enriched pathways were those related to ‘phenylpropanoid biosynthesis’, ‘plant pathogen interactions’, ‘starch and sucrose metabolism’ etc. Of these, the pathway containing the most DEGs was ‘plant pathogen interactions’, followed by ‘starch and sucrose metabolism’ and ‘phenylpropanoid biosynthesis’. Interestingly, the GO analysis of DEGS suggested different groups, with most DEGs being involved in the biological processes ‘responses to stimuli’, followed by ‘responses to stress and chemicals’. These three biological processes were also the most significant of the enriched GO terms, with a Q value of 1e−07. In the cellular components and molecular functions categories, the most enriched entries included ‘integral component of membrane’, ‘ADP binding’, and ‘oxidative activation of various proteases’. Venn plot analysis showed that eight genes were differentially expressed in six groups, and that the functions of these eight genes were mainly related to ‘reproduction’, ‘defense mechanisms’, ‘inorganic ion transport and metabolism’, ‘response to acid chemical’, and ‘carbohydrate transport and metabolism’ (Fig. 6c and d; Supplementary Data Table S9). DEG analysis showed that two of the four candidate genes (*Rhdel02G0243600*, *Rhdel08G0220700*) related to bark roughness and identified using GWAS analysis had significantly different expression levels in *R. delavayi* and *R. irroratum*. This is further evidence that the candidate genes obtained by GWAS analysis were related to the rough cracked and smooth non-cracked traits of *Rhododendron* bark (Fig. 6e).

To further verify the results of transcriptome analysis, RT–qPCR was performed on the obtained candidate genes. The expression of candidate genes in different bark phenotypes was detected by the non-specific SYBRGreenI dye method (Supplementary Data Table S10). The results of RT–qPCR showed that the expression levels and trends of the four candidate genes were consistent with the transcriptome sequencing (Supplementary Data Fig. S9). The results of transcriptome analysis and RT–qPCR showed that the expression of candidate genes obtained by GWAS was significantly different in *R. delavayi* perennial branches and twigs, and *R. irroratum* perennial branches and twigs.

## Discussion

### Identification of SNPs associated with variant characters

We found 512 488 SNPs located in the CDS, and that non-synonymous mutations accounted for 50.58% of total SNPs and were the largest single type of variant site. Compared with major crop plants such as rice and wheat, *Rhododendron* is only in the initial stages of domestication, and its SNP density is therefore significantly higher. The genome-wide average SNP density in our studied *Rhododendron* taxa was 124 bp per SNP, while in rice and wheat the density is 268 bp and 46 kb per SNP, respectively [25, 26].

The genetic diversity in our *Rhododendron* populations was higher than that seen in crop plants, and LD also decayed faster in *Rhododendron* than in other crops. For example, the LD decay distance is ~100 kb in rice and ~ 2 kb in maize [27], while the decay distances of *R. delavayi*, *R. irroratum*, and *R. agastum* are 150, 132, and 64 bp, respectively. In the horticulturally important flowering tree *Prunus mume*, the LD block coverage of SNPs related to target traits reached 11.1–11.8 Mb, while in *Rhododendron* the maximum LD block coverage was only 62.97 kb [9].

These results also suggest that there is a higher degree of individual diversity and more obvious intraspecies differentiation in *R. agastum* than in our other two study taxa. The mean *F*_ST_ values were 0.35, 0.11, and 0.12 for *R. delavayi* and *R. irroratum*, *R. delavayi* and *R. agastum*, and *R. irroratum* and *R. agastum*, respectively. This suggests that *R. delavayi* and *R. irroratum* are two independent species, as they have the highest degree of genetic differentiation among the three species of *Rhododendron*, a result which is consistent with the PCA and population structure analyses. The mean *F*_ST_ values of *R. agastum* within *R. delavayi* and *R. irroratum* were between 0.05 and 0.15, indicating that *R. agastum* was moderately differentiated from *R. delavayi* and *R. irroratum*, but was more differentiated from *R. irroratum*. The mean θπ of *R. agastum* was 0.0026 and this taxon had the highest degree of intraspecific individual diversity among the three studied *Rhododendron* taxa. A greater degree of differentiation between the natural race of einkorn (β) and the hybrid domesticated variety has been observed, with an *F*_ST_ value between the β race and domesticated einkorn reaching 0.31 [28].

PCA analysis divided *R. delavayi* and *R. irroratum* obviously into two groups, while the individuals within *R. agastum* were also divided into two groups, which was consistent with the *F*_ST_ results and with previous research by Marczewski *et al*. [29]. There was a high degree of individual variation and intra-population differentiation in *R. agastum*. The value of *K* with the minimum cross-validation error was 3, and our 260 *Rhododendron* samples were then divided into three subgroups. Individuals of *R. delavayi* gathered in a separate cluster, as did individuals of *R. irroratum*, and the heterozygosity of individuals within each of these clusters was not high. However, the heterozygosity of individuals in the *R. agastum* cluster was high. Analysis of the population structure of *R. agastum* suggested that further ancestor populations besides *R. delavayi* and *R. irroratum* have contributed to the *R. agastum* genome. Therefore, the reason why *R. agastum* has higher individual diversity and more phenotypes than are observed in *R. delavayi* and *R. irroratum* could be because *R. agastum* has multiple wild *Rhododendron* parents, which is also consistent with the results of Zhang *et al*. [30].

The average genetic distance between *R. delavayi* and *R. agastum* was 0.82, while the average genetic distance between *R. irroratum* and *R. agastum* was only 0.78, indicating that *R. delavayi* and *R. agastum* are closely related. This result is consistent with the results obtained by Zheng *et al*. using nuclear gene spacer sequence ITS and chloroplast gene fragment sequencing [31].

### Candidate genes identified using GWAS analysis potentially contribute to bark traits

Due to the complex genetic background of *Rhododendron*, a variety of horticultural traits may be greatly influenced by the external environment. We selected bark roughness for study, as it is less influenced by the environment, and as a peripheral protective structure of the trunk, bark can also help plants resist infection by external pathogens.

Bark can be divided into outer epidermis, a pericarp composed of cork, cork cambium, and cork inner layer, and phloem [32]. In the early stage Marczewski *et al*. [29] assessed the roughness of the bark as a qualitative trait, and divided it into ‘rough’, ‘smooth’, and ‘intermediate’. In this study, we divided the roughness of the bark into rough and smooth, as, based on the varying growth cycles of the samples, we believe that intermediate is only a transitional form of the roughness of the bark, and that intermediate trees will eventually become either rough or smooth.

The results of GO and KEGG enrichment analysis showed that the three *Rhododendron* taxa studied here may regulate the degree of water loss by regulating the expression of genes related to waxy and fatty acid synthesis in the bark. However, the bark phenotypes of these three taxa are significantly different [33]. The FKBP17-1 encoded by *Rhdel02G0243600* is a member of the FKBP (FK506 binding protein) immunophilin family and belongs to the PPIase (peptidyl prolyl *cis*-*trans* isomerase) superfamily of immune-suppressive drugs. In plants, FKBPs are ubiquitous and involved in various biological and physiological processes, including plant germination, development, stress responses, and hormonal signaling. FKBP42 can regulate the multidrug resistance-like transporter family AtPGP1/AtPGP19 in *Arabidopsis* to affect auxin export. At the same time, *fkbp42* and *fkbp72* mutant plants had reduced cell elongation, and *fkbp42* mutant plants were dwarf and non-directional [34]. *fkbp72* mutant plants are defective in cell division and the mutation affects the synthesis of very long-chain fatty acids (VLCFAs), an important component of plant epidermis wax [35]. These results suggest that FKBP immunophilins play an important role in affecting plant growth and development and related signal transduction.

Carbon catabolite repression4-negative (CCR4-NOT), encoded by *Rhdel04G0017100*, was first identified in *Saccharomyces cerevisiae* [36]. It is a unique, essential, and conserved multi-subunit complex that has many different cellular functions in regulating gene expression. Although there are no direct results demonstrating that the CCR4-NOT complex is associated with rough cracking and smooth non-cracking of bark, the conserved subunit NOT4 contained in the CCR4-NOT complex has been shown to function as a ring-domain E3 ubiquitin ligase to regulate the mass formation of fibers. The composition of this complex varies among species, but there are seven highly conserved subunits considered to be the core subunits [37]. The NOT4 subunit is a RING domain E3 ubiquitin ligase that polyubiquitinates protein substrates, targeting them for degradation [38]. In cotton, multiple genes (*GhHUB2*, *GhRING1*, *GB_A03G0335*) encoding E3 ubiquitin ligases have been reported to be associated with cotton fiber mass formation [39, 40].

Aspartic protease (AP), encoded by *Rhdel07G0079700*, is a very important subfamily member of the proteolytic enzyme family, which plays an important role in protein processing and degradation, plant senescence, and programmed cell death [41, 42]. Cao *et al*. identified a total of 67 AP genes in *Populus trichocarpa* (*PtAP*), and semi-qRT–PCR analysis further determined that at least 10 *PtAP*s were highly or preferentially expressed in the developing xylem [43]. Moreover, the *PtAP66* promoters were observed to be active only in interfascicular fiber cells, which are found in stems and are involved in shoot stability. *PtAP66* and *PtAP17* also had a function in wood formation. This was the first time that *AED-like* genes were reported to be involved in the formation of wood. We compared the protein sequence encoded by *Rhdel07G0079700* with the poplar protein sequence used by Cao *et al*. and found that the protein sequence encoded by *Rhdel07G0079700* could be matched to 42 AP protein sequences, with an e value of 3.34e−15.

GDSL2, encoded by *Rhdel08G0220700*, belongs to the GDSL lipase family. This type of lipase is widely found in prokaryotes and eukaryotes and plays an important role in plant growth and development, organ morphogenesis, secondary metabolism, and responses to stress [44, 45]. Park *et al*. compared the mutant *wdl1* with wild-type rice and found that the mutant showed high water loss, an abnormal waxy structure, loose cuticles, and uneven epidermal cell wall thickness [46]. Similarly, Girard *et al*. identified tomato GDSL1 from dewaxed tomato peel and found that GDSL1 is a protein necessary for the keratinization of the peel, and can participate in the extracellular deposition of cuticle keratin polyester of tomato fruits [47]. The deletion of its coding gene leads to thinning of the fruit epidermis and reduction of keratinization, resulting in cracking of the tomato fruit peel. Auxin-regulated GDSL lipases have also been identified in *Arabidopsis*. One set is required for suberin synthesis, while the other can drive suberin degradation. These results showed that GDSL lipases have key roles in suberization and driving root suberin plasticity [48].

Although we have not yet elucidated the mechanism by which the four candidate genes identified in the GWAS analysis cause the significant differences in the roughness of the bark in different *Rhododendron* taxa, we sampled different species and growth stages of *Rhododendron*, and transcriptome sequencing analysis and RT–qPCR further confirmed that there were indeed significant differences in the expression levels of *Rhdel02G0243600* and *Rhdel08G0220700* in *Rhododendron* taxa and growth stages.

**Figure 7:**
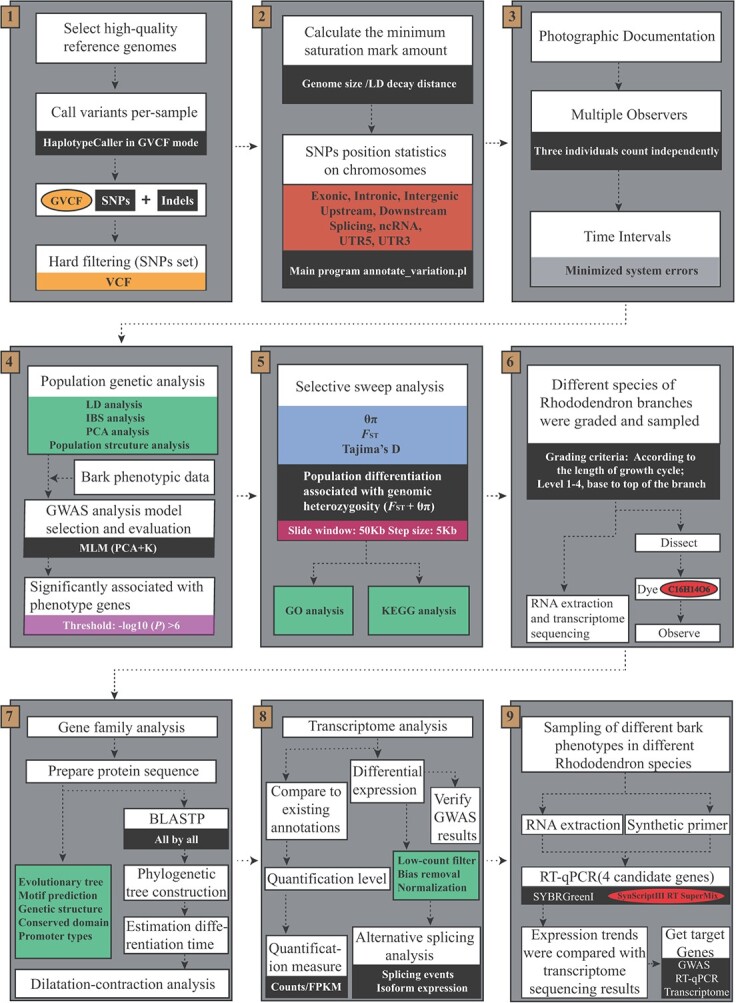
The analytical method and flowchart used in the research.

In addition, we developed a set of procedures for mining and studying genes related to important horticultural traits of wild rhododendrons by combining GWAS, transcriptome, RT–qPCR, and other methods, which can provide references for the development of wild rhododendron germplasm resources, identification of rhododendrons, and shortening the breeding time of new varieties (Fig. 7).

## Materials and methods

### Sampling and phenotyping

The samples of *R. delavayi*, *R. irroratum*, and their hybrid descendant, *R. agastum*, were all collected in Baili Scenic Reserve in western Guizhou, China (27.226–27.234 N, 105.848–105.856 E, altitude 1600–1700 m), during the flowering season of the two species (*R. delavayi*, *R. irroratum*). The sampled individuals were quickly divided into the two ‘pure’ species *R. delavayi* and *R. irroratum* and hybrids (*R. agastum*) based on flower color, bark structure, leaf indumentum, and stamen hairs. Bark phenotypes of individuals were assessed directly in the field, and each individual bark sample was measured nine times. The bark of each sampled individual was photographed.

### DNA isolation and genome resequencing

Individual leaves from each sample were used for DNA extraction. Resequencing was performed according to the standard protocol provided by Illumina. The original image data files obtained by high-throughput sequencing were converted into raw reads using base-calling analysis and stored as FASTQ files. The raw reads were then filtered according to the following criteria: (i) reads with adapters were removed; (ii) reads with an N content of >10% were removed; and (iii) reads with base quality <10 and >50% were removed. The quality and type distribution of nucleobases were analyzed, and after filtering 2000 high-quality reads were randomly selected and compared with the NT library using BLAST software. Finally, 8067.62 Mb clean reads were obtained.

### RNA extraction and transcriptome sequencing

Older specimens of *R. delavayi* and *R. irroratum* were selected for sampling, and the branches were divided into four categories: *R. delavayi* perennial branches (lower branches) and *R. delavayi* twigs (upper branches), and similarly, *R. irroratum* perennial branches and twigs. After dissection, the cambium was removed and other tissues were snap-frozen in liquid nitrogen and subjected to transcriptome sequencing. There were three replicates for each category, giving a total of 12 samples. Samples that passed the quality inspection were used in the construction of a cDNA library. The effective concentration of the library was quantified using Q-PCR, and sequenced on an Illumina platform, after ensuring that the quality of the library reached the standard. The raw data were filtered, with connectors and low-quality reads being removed, and the clean reads were saved in FASTQ format.

### Detection of population SNPs

False-positive SNPs were filtered out before subsequent analysis. In order to exclude SNP-calling errors caused by incorrect mapping, we used hard filtering based on variant information to filter the SNPs. To remove SNPs near indels and indels with an interval of <10 bp, we used the software BCFtools (version 1.2). After processing, the average sequencing depth of the samples was calculated, and this was used to inform multi-allelic and average sequencing depth filtering on the variants. The software VCFtools (version 0.1.13) was used to set the filtering parameters to --minDP 3 --min-alleles 2 --max-alleles 2, then to QD < 2.0, SOR > 3.0, FS > 60.0, MQ < 40.0, MQRankSum < -12.5, ReadPosRankSum < -8.0, according to the official hard filtering standard of GATK software. The remaining SNPs were further filtered based on the sample deletion rate and site deletion rate using the software PLINK (version 1.9), setting the parameters to --mind 0.1 --geno 0.05 --maf 0.05 --hwe 0.001. After filtering, only high-quality SNPs [depth ≥3, biallelic loci, missing rate ≤10% of samples in the population, locus missing rate ≤5%, minor allele frequency (MAF) ≥0.05] were reserved for subsequent analysis.

### Phylogenetic and population structure analysis

The software SNPhylo (version 2018-09-01) was used to build a maximum likelihood tree with DNAML (https://github.com/thlee/SNPhylo). Bootstrap values were derived from 1000 replicates and 260 sample phylogenetic tree diagrams were drawn online using ITOL (https://itol.embl.de). The population genetic structure was assessed using Admixture v1.3.0 (http://dalexander.github.io/admixture/download.html). The number of assumed genetic clusters *K* ranged from 1 to 10. Cross-validation errors were checked using grep; a *K* value corresponding to the smallest cv error was selected. We evaluated the genetic structure by using PLINKv1.9 software (https://www.cog-genomics.org/plink2).

### Population genetics and linkage disequilibrium analysis

Fixation statistics (*F*_ST_) and nucleotide diversity (θπ) were calculated in VCFtools v0.1.13. The sliding window size and sliding windows step were 10 and 5 kb respectively. PopLDdecay (version 3.41) was used to calculate the VCF file of 260 samples to obtain the *r*^2^ value. Linkage disequilibrium analysis of SNPs in specific regions was conducted using PLINK v1.9. We used LDBlockShow (version 1.33) and Haploview (version 4.1) to draw the LD block of a specific region.

### GWAS analysis

A total of 5 328 800 SNPs were used in the genome-wide association analysis of bark phenotypes (MAF ≥0.05; missing rate ≤0.05; depth ≥3). To correct for SNP false positives due to high differentiation between populations and structural changes of genetic populations in the natural population sampling, we included the results of PCA and kinship analysis as an association matrix. Bark phenotypes were associated with SNPs using various association models in the software package GAPIT (version 3.0) (https://github.com/jiabowang/GAPIT). The correlation models used include the general linear model (GLM), efficient mixed linear model (MLM), fixed and random model circulating probability unification (FarmCPU), compressed mixed linear model (CMLM), fixed and random model circulating probability unification (SUPER), optimized compressed mixed linear model (enriched CMLM, ECMLM), and the Bayesian-information and linkage-disequilibrium iteratively nested keyway (BLINK).

The model used is described using the following equation:

$$ y= X\beta + S\alpha + Qv+ Zu+e $$

Where y is a vector of phenotypic observation; β is a vector of fixed effects other than SNP or population group effects; α is a vector of SNP effects (QTN); v is a vector of population effects; u is a vector of polygene background effects; e is a vector of residual effects; Q is a matrix from STRUCTURE relating y to v; And X, S and Z are incidence matrices of 1s and 0s relating y to β, α and u, respectively. The effect values of our genetic markers were tested using *F* tests and corrected for multiple testing using Bonferroni correction. Only the most obvious SNP peak in the Manhattan plot was chosen as the candidate SNP. Meanwhile, to estimate the differences between the observed and predicted values of each quantitative trait, all Manhattan results were validated with QQ plots.

### Gene family analysis

We used pfscan to determine the PF number of each candidate gene and the HMM files were then downloaded from the PFAM database (https://www.ebi.ac.uk/interpro). The gene family members of the candidate genes in *R. delavayi* and *R. irroratum* were identified using HMMER (version 3.1b2). The target sequences were extracted using the software BEDtools (version 2.25.0). A phylogenetic tree was constructed using the software MEGA (version 11) for protein sequences with e-value less than 1e−5 identified previously by the software HMMER. Motif predictions of candidate gene families were performed using the MEME online website (https://meme-suite.org/meme/). Conserved domain analysis of candidate gene families was performed using NCBI Batch CD-Search (https://www.ncbi.nlm.nih.gov/). Gene structure analysis of candidate gene families was performed using TBtools (version 1.098). The reference genome annotation and the gene family member ID to which the candidate gene belonged were then used as input to obtain the intron, exon, CDS, and UTR positions of the target genes. Promoters of the candidate gene families were predicted using the online site PlantCARE (http://bioinformatics.psb.ugent.be/webtools/plantcare/html/). Biopython (version 1.79) was used to predict the physicochemical properties of the target protein sequences.

OrthoFinder (version v2.5.4) was used for the gene family clustering analysis. BLASTP all-by-all search (significance threshold e-value = 10e−3) was used to obtain homologous genes, and then BLAST bit scores were normalized based on gene length and phylogenetic distance. The orthogroup graph was constructed using RBNHs (reciprocal best length-normalized hit) to determine the threshold of sequence similarity of homologous groups. After Markov clustering (MCL) based on the BLASTP results, the script cafetutorial_mcl2rawcafe.py was used to convert the results into the file format required by CAFE (version 4.2.1). RAxML (version 8.2.12) was used to construct phylogenetic trees using the maximum likelihood method, and the trees were integrated in ASTRAL (version 5.7.8). The batch run script for r8s was built in cafetutorial_prep_r8s.py, and the ultrametric tree was extracted and run in CAFE, with the *P* value set to 0.01 and other parameters to default. The IDs of significantly expanded or contracted gene families were extracted from the file global_cafe.out.summary_fams.txt.

### Transcriptome differential expression analysis

Transcriptome differential expression analysis was performed after sequence alignment using the *R. delavayi* genome as the reference genome. Sequence alignment was performed in HISAT2 (version 2.0.4). The reference genome was first indexed, and this was then used to perform paired-end sequencing alignment. The obtained SAM files were sorted and converted into BAM files. The transcripts were assembled using the software StringTie (version 1.3.4d) with reference to the genome annotation files as input files. The results were visualized using the software IGV (version 2.8.13). The expression levels of sample transcripts were measured with the maximum flow algorithm and FPKM (fragments per kilobase of transcript per million fragments mapped). Genes with significant differences in their expression levels in *Rhododendron* taxa with different types of bark were screened out. Differential expression gene analysis between sample groups used data were counts. Software DESeq2 (version 1.6.3) was used to transform counts, and the screening criteria were fold change ≥2 and FDR ≤0.01. Enrichment analysis of differentially expressed genes was then conducted using the COG (http://www.ncbi.nlm.nih.gov/COG/), KOG (ftp://ftp.ncbi.nih.gov/pub/COG/KOG/kyva), GO (http://www.geneontology.org/), KEGG (http://www.genome.jp/kegg/) and other databases.

### Relative quantitative detection by qPCR

The RNA samples of *R. delavayi* perennial branches and twigs and *R. irroratum* perennial branches and twigs extracted before were used as templates. After the quality inspection of the RNA, the integrity of the RNA was good and the OD_260_/OD_280_ of the total RNA was between 1.8 and 2.1, so the RNA could be used for subsequent experiments. After the CDS sequence of the target genes had been extracted using gffread (version 0.12.8), the primer was designed (Supplementary Data Table S11). Reverse transcription amplification was performed using SynScript^®^ III RT SuperMix for qPCR, adding the following components (RNA template, 4 μl; 5 × gDNA Remover Mix, 2 μl; and RNase-free water, up to 10 μl) to the samples. The mixture was incubated at 42°C for 2 min and at 60°C for 5 min. It was then quickly placed on ice and cooled. After a short centrifugation, the following components were added: Rnasin, 1 μl; 5 × SynScript^®^ III RT SuperMix, 4 μl; and RNase-free water, up to 20 μl. After mixing, the samples were incubated at 25°C for 10 min, 50°C for 30 min, and 85°C for 5 min. The cDNA product obtained by reverse transcription was diluted 5-fold and used as qPCR template for amplification using ArtiCanCEO SYBR qPCR Mix. The pre-denaturation temperature was set at 95°C for 5 min. Cycle stage settings were as follows: temperature 95°C, 15 s; 60°C, 20 s; 72°C, 20 s; number of cycles, 40. Melting stage temperature was 95°C, four cycles.

## Acknowledgements

We thank Hong Yang for phenotypic data collection. This research was supported by the ZIAT-Special Project (ZJKJT-2022-01) and the Science and Technology Talent and Platform Program of Yunnan Province (202105 AC160017), the Yunnan Province Agriculture joint special project (202101BD070001-010), and the China Agriculture Research System of MOF and MARA (CARS-23-G56).

## Author contributions

C.Z. and J.W conceived the study and participated in revision and discussion; Q.Y., Q.L., Y.Z., and Y.J. executed formal analysis, methodology and project administration; Z.W., Y.H., and Y.J. helped in investigation, data curation and resources; L.Z. conducted experiments; Q.Y., L.Z., and C.Z. drafted the manuscript with significant participation of Y.M. All authors read and approved the final manuscript.

## Data availability

The authors confirm that the data supporting the findings of this study are available within the article and its supplementary materials.

## Conflict of interest

The authors have no conflicts of interest to declare.

## Supplementary data

Supplementary data is available at *Horticulture Research* online.

## Supplementary Material

Web_Material_uhae008
