# Supplementary material for: Genome and GWAS analysis identified genes significantly related to phenotypic state of Rhododendron bark
Source: Hortic Res. 2024 Jan 10;11(3):uhae008. doi: 10.1093/hr/uhae008 (PMC10939351; doi:10.1093/hr/uhae008)
Supplement: Web_Material_uhae008 [file web_material_uhae008.zip › Supplementary Fig. 1.pdf]

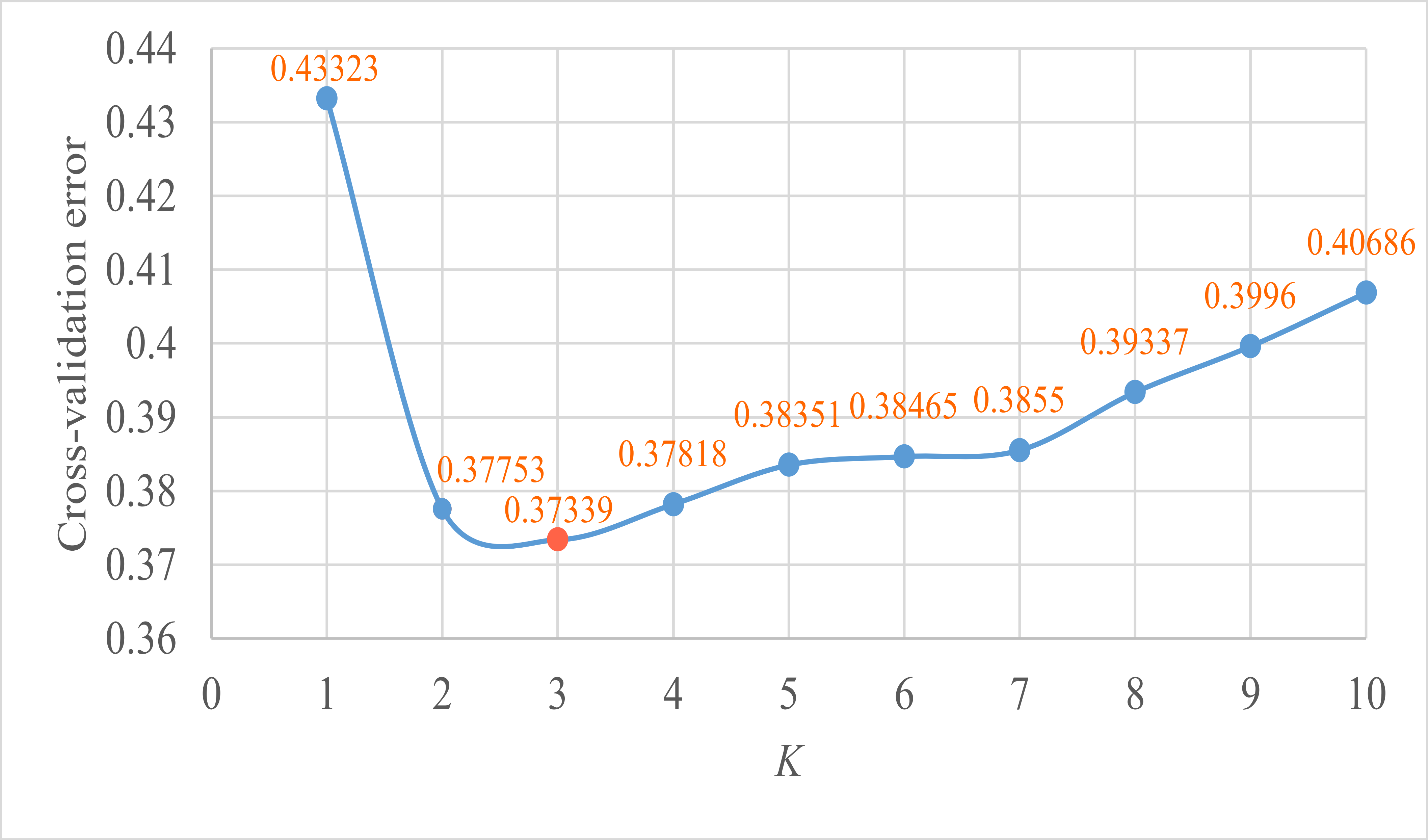

Figure S1.Three taxa of *Rhododendron K*-fold cross validation. The software ADMIXTURE (version: v1.3.0) is used to set the *K* value range of 1-10. When *K* is equal to 3, the cross-validation error is minimum.
