# Supplementary material for: Genome and GWAS analysis identified genes significantly related to phenotypic state of Rhododendron bark
Source: Hortic Res. 2024 Jan 10;11(3):uhae008. doi: 10.1093/hr/uhae008 (PMC10939351; doi:10.1093/hr/uhae008)
Supplement: Web_Material_uhae008 [file web_material_uhae008.zip › Supplementary Fig. 2.pdf]

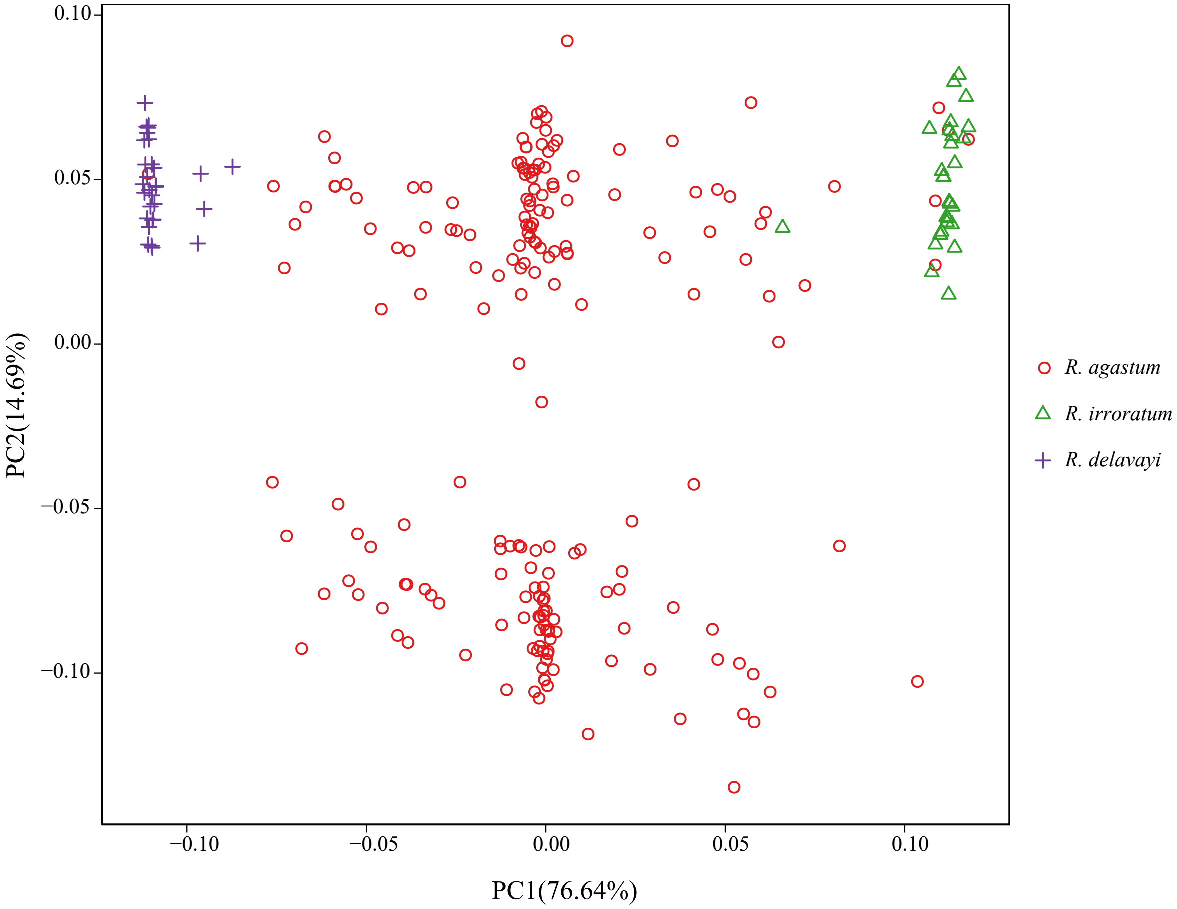

Figure S2. Principal component analysis of three taxa of *Rhododendron*. The number of principal components was set to 3, the explanation proportion of PC1 and PC2 for SNPs phenotypic variation was determined.
