# Supplementary material for: Genome and GWAS analysis identified genes significantly related to phenotypic state of Rhododendron bark
Source: Hortic Res. 2024 Jan 10;11(3):uhae008. doi: 10.1093/hr/uhae008 (PMC10939351; doi:10.1093/hr/uhae008)
Supplement: Web_Material_uhae008 [file web_material_uhae008.zip › Supplementary Fig. 3.pdf]

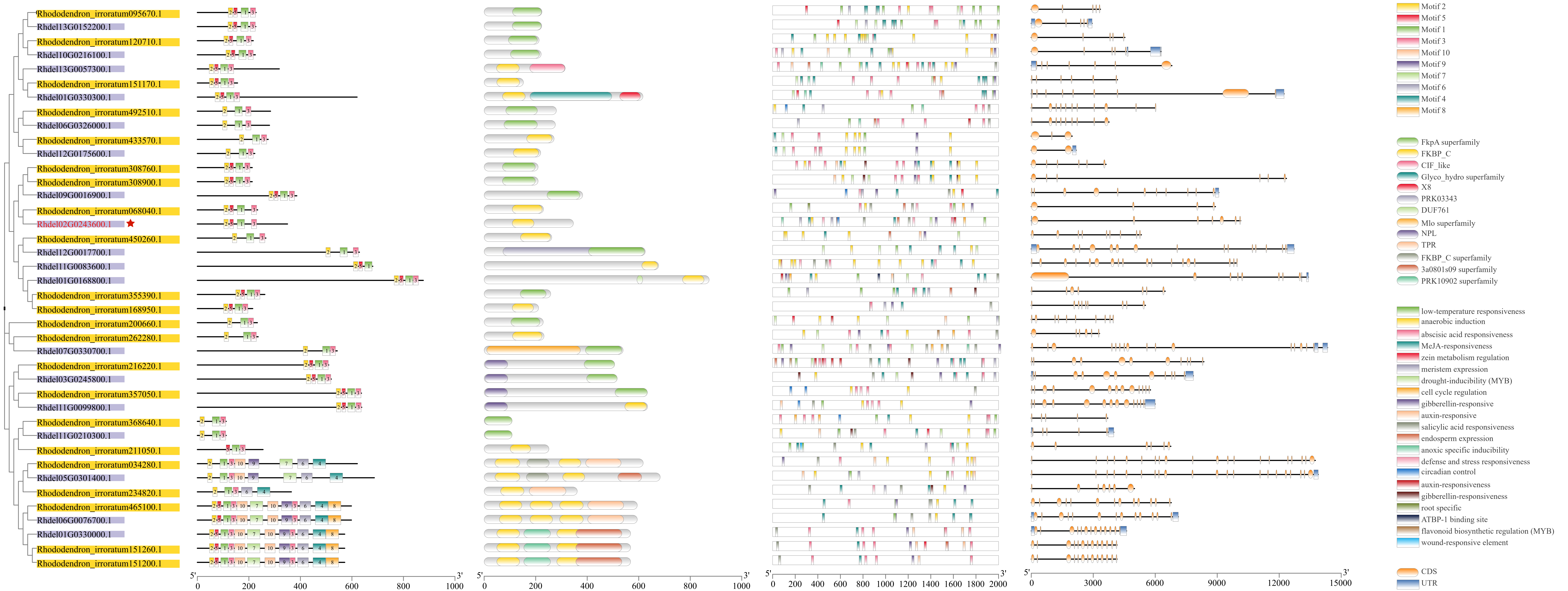

Figure S3. The Gene family analysis of candidate gene *Rhdel02G0243600*. From left to right are the evolutionary trees constructed by the identified *R. delavayi* and *R. irroratum* gene family members, motif analysis, conserved domain analysis, promoter type prediction and gene structure analysis. Candidate gene is marked with asterisks.
