# Supplementary material for: Genome and GWAS analysis identified genes significantly related to phenotypic state of Rhododendron bark
Source: Hortic Res. 2024 Jan 10;11(3):uhae008. doi: 10.1093/hr/uhae008 (PMC10939351; doi:10.1093/hr/uhae008)
Supplement: Web_Material_uhae008 [file web_material_uhae008.zip › Supplementary Fig. 7.pdf]

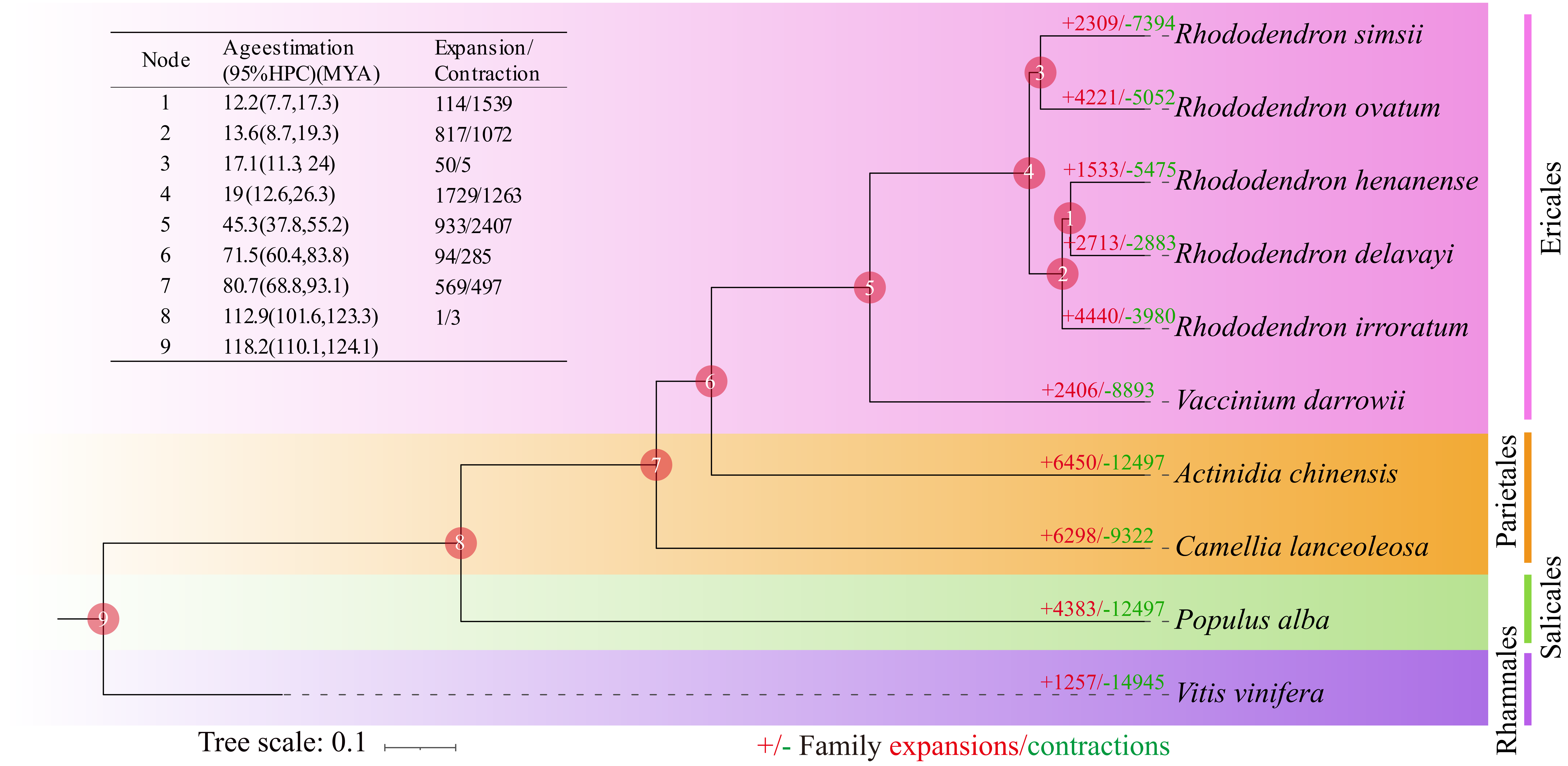

Node	Ageestimation (95%HPC)(MYA)	Expansion/ Contraction
1	12.2(7.7,17.3)	114/1539
2	13.6(8.7,19.3)	817/1072
3	17.1(11.3, 24)	50/5
4	19(12.6,26.3)	1729/1263
5	45.3(37.8,55.2)	933/2407
6	71.5(60.4,83.8)	94/285
7	80.7(68.8,93.1)	569/497
8	112.9(101.6,123.3)	1/3
9	118.2(110.1,124.1)	

Figure S7. Analysis of gene family contraction and expansion and estimation of differentiation time in 10 species. The MCMCTREE program in PAML (version:v4.9i) software package was used to estimate the differentiation time. Two calibration points for fossil time *Vitis vinifera* and *Populus alba* (109.0-123.5MYA). *Vaccinium darrowii* and *Rhododendron ovatum* (40.8-82.9 MYA).
