# Supplementary material for: Genome and GWAS analysis identified genes significantly related to phenotypic state of Rhododendron bark
Source: Hortic Res. 2024 Jan 10;11(3):uhae008. doi: 10.1093/hr/uhae008 (PMC10939351; doi:10.1093/hr/uhae008)
Supplement: Web_Material_uhae008 [file web_material_uhae008.zip › Supplementary Fig. 8.pdf]

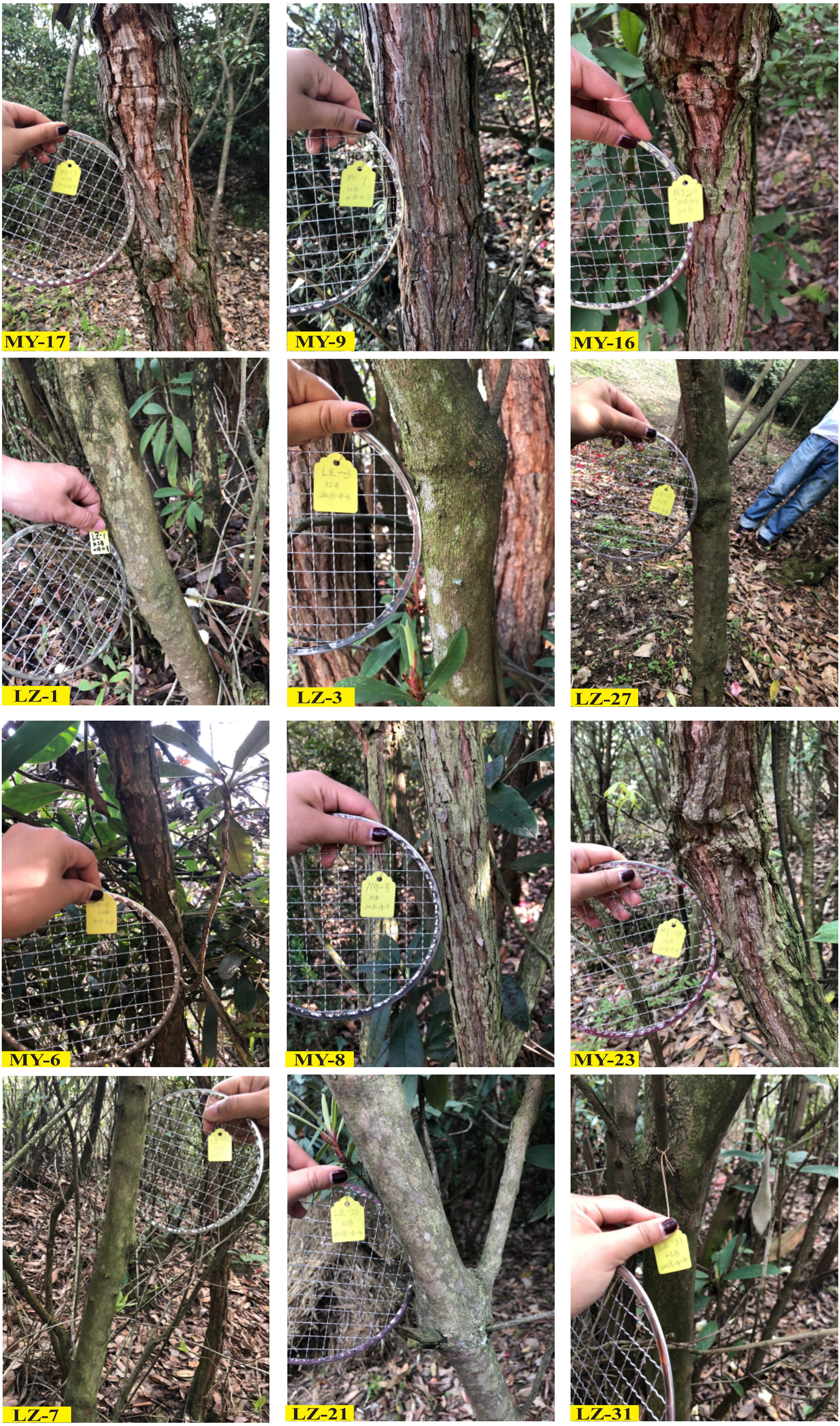

Figure S8. **Bark phenotypes of *R. delavayi* and *R. irroratum*.** The label at the bottom left of the picture is the sample number. MY: *R. delavayi*; LZ: *R. irroratum*.
