# Supplementary material for: Genome and GWAS analysis identified genes significantly related to phenotypic state of Rhododendron bark
Source: Hortic Res. 2024 Jan 10;11(3):uhae008. doi: 10.1093/hr/uhae008 (PMC10939351; doi:10.1093/hr/uhae008)
Supplement: Web_Material_uhae008 [file web_material_uhae008.zip › Supplementary Fig. 9.pdf]

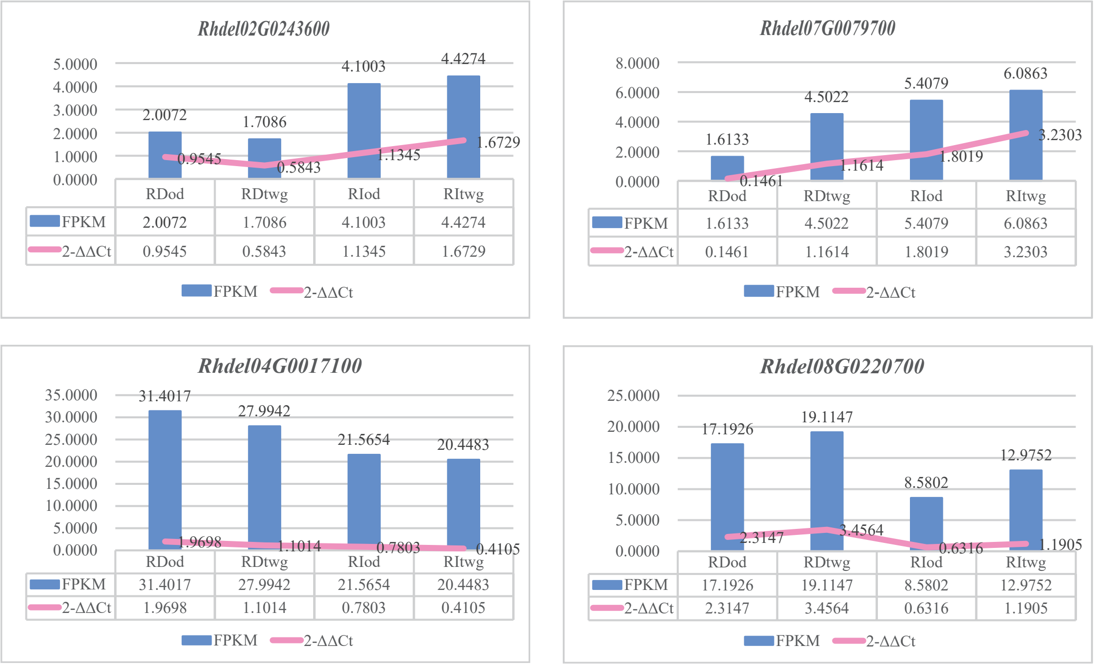

Figure S9. Expression level and trends of candidate genes. The blue bars are the candidate genes expression level (FPKM) obtained by transcriptome sequencing; The pink lines are the expression level (2-ΔΔCt) of candidate genes obtained by RT-qPCR.
