# Supplementary figures and images for: Genome and GWAS analysis identified genes significantly related to phenotypic state of Rhododendron bark
Source: Hortic Res. 2024 Jan 10;11(3):uhae008. doi: 10.1093/hr/uhae008 (PMC10939351; doi:10.1093/hr/uhae008)

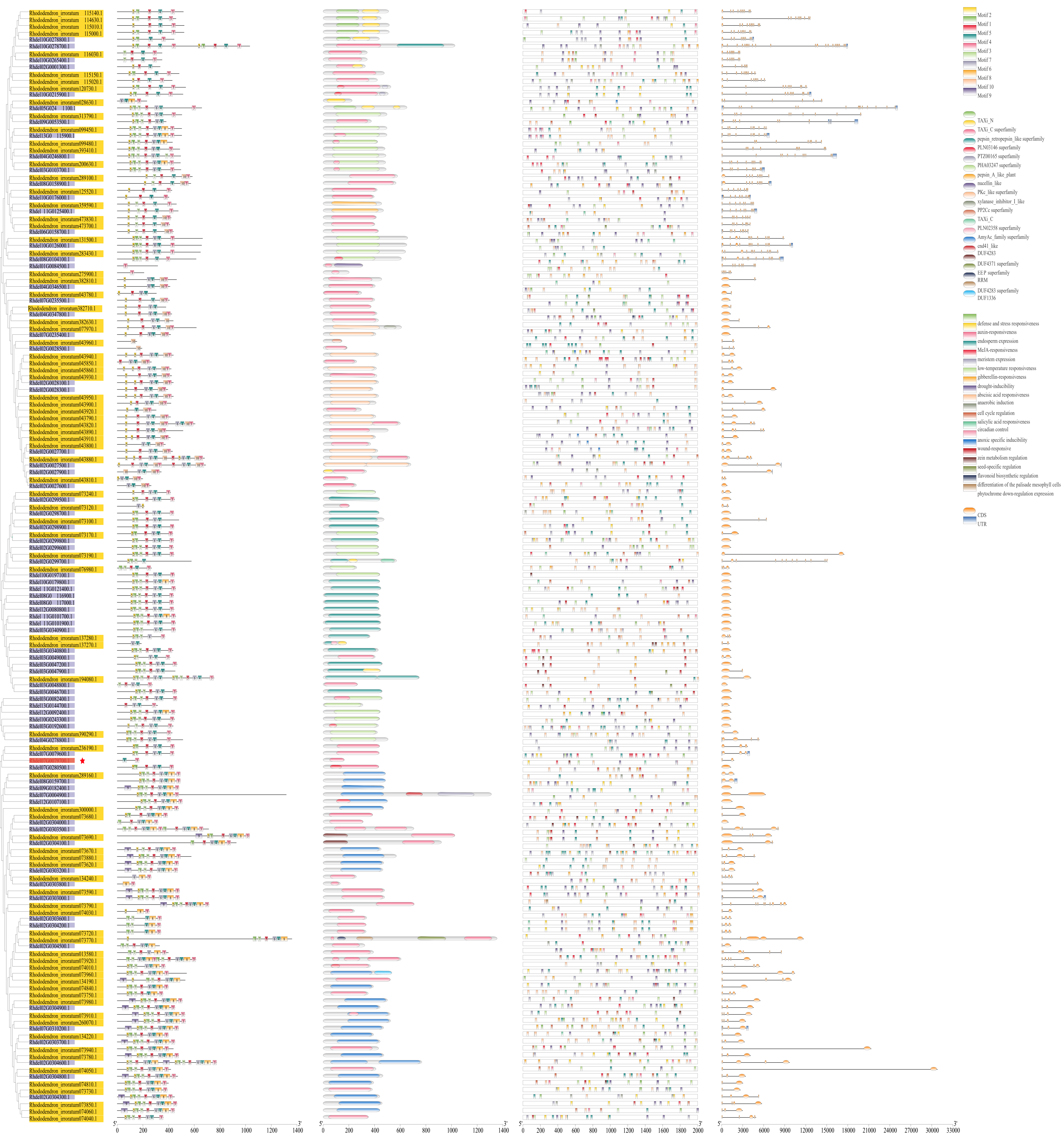

Supplement: Web_Material_uhae008 [file web_material_uhae008.zip › Supplementary Fig. 5.pdf]
